# Examining the Interaction between *Phytophthora sojae* and Soybean Cyst Nematode on Soybean (*Glycine max*)

**DOI:** 10.3390/plants11040560

**Published:** 2022-02-21

**Authors:** Rawnaq N. Chowdhury, Paul N. Okello, Emmanuel Byamukama

**Affiliations:** 1 Small Grains and Potato Germplasm Research Unit, United States Department of Agriculture, Agricultural Research Service, West Aberdeen, ID 83210, USA; 2 Department of Agronomy, Horticulture and Plant Science, South Dakota State University, Brookings, SD 57007, USA; paul.okello@sdstate.edu (P.N.O.); emmanuel.byamukama@sdstate.edu (E.B.)

**Keywords:** *Phytophthora*, SCN, soybean

## Abstract

*Phytophthora sojae* and soybean cyst nematode (SCN) are important pathogens of soybean. Although these pathogens infect soybean roots, there is limited evidence of any interaction between them. The objective of this study was to examine the interaction between SCN and *P. sojae* on soybean in the greenhouse. Seeds of four soybean cultivars (Jack, Surge, Williams 82, Williams) were pre-germinated and placed in cone-tainers (Stuewe and Sons Inc., Tangent, OR, USA), containing a steam pasteurized sand-clay mixture. The experiment was set up in a completely randomized design with five replications and performed twice. Two *P. sojae* isolates were used in this study that represented two different virulence pathotypes (simple and complex pathotypes). For each isolate, soybean plants were not inoculated, inoculated with one of the treatments—SCN, *P. sojae*, and combination of *P. sojae* and SCN. After 35 DOI, stem length, root length, plant weight, root weight, lesion length, and SCN population were recorded. On all soybean cultivars with different types of incomplete resistance, the complex pathotype (PS-15-TF3) influenced the lesion length (mm) in the presence of SCN. However, the SCN population was reduced by both complex and simple pathotypes of *P. sojae*. This suggests that use both SCN and *P. sojae* resistance cultivars, can manage the disease complex and reduce soybean yield loss.

## 1. Introduction

In South Dakota, soybean *Glycine max* L. (Merr.) is an important crop for farmers. In 2020, the production of soybean in South Dakota accounted for an estimated five million hectares planted, worth approximately $2.37 billion in revenue according to the 2020 market values for soybean [https://www.nass.usda.gov/Quick_Stats/Ag_Overview/stateOverview.php?state=SOUTH%20DAKOTA/ Accessed on 16 February 2022]. Among the factors limiting soybean production in South Dakota, the soybean cyst nematode, (*Heterodera glycines* Ichinohe, SCN) and *Phytophthora sojae* Kaufmann and Gerdemann (the causal pathogen of *Phytophthora* root and stem rot) are very important. In 2013 and 2014, randomly selected commercial soybean fields (250 fields covering 28 counties in 2013 and 200 fields covering 24 counties in 2014) were surveyed for SCN and *P. sojae* by testing soil samples collected from these fields for the presence of the two pathogens. The number of SCN eggs and J2 were counted, and soils were tested for presence of *P. sojae* [1]. The survey was supported by soybean checkoff funds from the South Dakota Soybean Research and Promotion Council (Sioux Falls, SD, USA). From the two years survey, it was determined that 25 fields (10 counties) in 2013 and 19 fields (14 counties) in 2014 were positive for both SCN and *P. sojae* [1]. These South Dakota fields that were confirmed for the co-presence of the two pathogens represented different soil textures; for example, most of the fields in the north-eastern counties had a fine textured (clay-loam) soil and fields in the southeastern counties had a coarse textured (sandy) soil (NRCS, https://websoilsurvey.sc.egov.usda.gov/ accessed on 12 November 2020). The two pathogens were identified mostly in fields with clay loam soil (NRCS, https://websoilsurvey.sc.egov.usda.gov/ accessed on 12 November 2020) and symptoms on soybean plants associated with the two pathogens were observed [2]. Currently, there is no information available on the yield loss due to the coexistence of SCN and *P. sojae* on soybean plants in these fields. However, it is possible that the soybean farmers are experiencing more yield losses from the two pathogens together as compared to losses that would result from either of the pathogens by itself.

In the United States *Phytophthora sojae* causes the major yield-limiting disease known as *Phytophthora* root and stem rot of soybean. The pathogen is known to infect soybean at all stages [3]. *Phytophthora* root and stem rot of soybean causes an approximate loss of $338 million annually to the United States producers according to the 2014 market values for soybean (data from the USDA—National Agricultural Statistics Service; https://www.nass.usda.gov/ accessed on 12 November 2020) [4]. In South Dakota, the yield losses associated with the disease are 4 to 6% of the crop yield every year [5]. To assess the pathogen diversity in *P. sojae* a virulence test is usually done by using a set of different soybean lines. There are several soybean lines (seven to 14 soybean lines) each of which contains one resistance gene (*Rps*) to *P. sojae* and a universally susceptible one (Williams) used to characterize *P. sojae* races or pathotypes [6,7]. To date, more than 55 pathogenic races have been described based on the compatible (susceptible) and incompatible (resistant) reactions on differential lines [6,8]. Depending on the previously described virulence formula [9] a race number was given to a pathotype of *P. sojae.* As new virulence gene combinations or pathotypes were continuously emerging in the pathogen the previously described race classification system become complicated [6]. Presently, pathotypes or virulence formulas are used to define virulence patterns based on reactions on a differential. From the surveys conducted to determine the pathotype of *P. sojae* population prevalent in the soybean production regions of South Dakota in 2013 and 2017, *P. sojae* pathotypes were identified that were virulent on all 13 soybean differentials [1]. Currently, *P. sojae* is managed by farmers using qualitative and quantitative resistance in commercial soybean varieties [6,10,11].

Under field conditions, among the factors that potentially have a role in affecting the disease severity caused by *P. sojae* on soybean, SCN is possibly important. The nematode has caused an approximate yield loss of 1.3 million metric tons, which accounts for $1 billion in revenue losses annually in the U.S. [12,13]. Given the fact that both pathogens are capable of infecting soybean roots, there are possibilities of interaction between the two pathogens, thus affecting the overall growth of soybean. Between the two pathogens, SCN is known to interact with soil-borne pathogens on soybean [14]. For example, McLean and Lawrence [15] have reported that soybean plants infected by both the Sudden Death Syndrome (SDS) fungus (*Fusarium virguliforme*) and SCN developed severe symptoms of SDS compared with plants inoculated only with *F. virguliforme*. As compared to plots inoculated with fungus only, the incidences of SDS symptoms were 35% and 18% higher than in plots containing both *H. glycines* and *F. solani.* A study by Diaz [16] observed significant greater root rot in soybean seedlings when soils were infested with both *Fusarium* and SCN. They also found more detrimental effect on root dry weight, root length, total surface area, and number of forks and tips in presence of both *Fusarium* and SCN that as compared to individual pathogen treatments. Frohning [17] also studied the interaction between *Rhizoctonia solani* and *Heterodera glycines,* but their study was not able to clearly detect the interaction between the two pathogens. Mycelial suspension of *R. solani* and SCN HG type 2.5.7. population at zero, low, and high levels were used for the infestation on resistant (S35-T9) and susceptible cultivars (S36-B6). With the increasing pressure of SCN the values for plant height, dry shoot weight, and dry root weight decreased in the treatment combinations. Adeniji et al. [18] studied the interaction between SCN HG type 0 (Race 3) and *P. sojae* pathotype represented by virulence formula 00001 (Race 1) on soybean and it was observed that plants of the susceptible soybean cv. Corsoy had more severe disease in the seedling in presence of both pathogens compared to when *P. sojae* was present alone. The SCN population was significantly reduced in the roots of susceptible cv. Corsoy due to *P. sojae* infection. Recently, Audette et al. [19] studied coinfection of soybean plants with *P sojae* and SCN and claimed that *P sojae* might indirectly influence SCN development but *P. sojae* resistance were not altered by the presence of SCN. 

To manage SCN, most farmers in the North Central United States, including in South Dakota, use soybean cultivars with resistance derived from PI 88788, Peking, or PI 437,654 [20,21]. In these commercial SCN resistant varieties, the genes *Rps*1a, *Rps*1c and *Rps*1k are commonly deployed in the form of incomplete resistance to manage *Phytophthora* root and stem rot in South Dakota [8]. However, shifts in *P. sojae* pathotypes have been implied in a recent study characterizing the pathotype diversity of *P. sojae* in commercial soybean fields in South Dakota and about 4% of the isolates were able to produce virulent reaction on all 13 soybean differentials [1]. In this study, we hypothesized that SCN presence can not only influence the lesion size caused by *P. sojae* pathotypes (on soybean cultivars with incomplete resistance to *P. sojae*), but the co-infection of the two pathogens can affect soybean growth during the infection process. To test the hypothesis, a *P. sojae* isolate (PS-15-TF3) that is virulent on all 13 soybean differentials (complex pathotype) is compared with a *P. sojae* isolate (PS-14-F14) representing Race 1 [showing virulent reaction on differential carrying *Rps*7, (simple pathotype)] during their individual interaction with SCN on four soybean cultivars with varying levels of incomplete resistance to SCN and *P. sojae* in the greenhouse. The specific objectives of this study were (i) to determine whether the interaction between SCN and *P. sojae* can affect soybean growth under greenhouse conditions; (ii) to evaluate the effect on lesion size caused by *P. sojae* pathotypes on soybean in presence of SCN in the greenhouse; and (iii) to evaluate the SCN development on soybean in the presence of *P. sojae* in the greenhouse. 

## 2. Results

### 2.1. Interaction of P. sojae and SCN on Soybean Growth

In the greenhouse, all soybean plants inoculated with *P. sojae* were observed to be symptomatic 12–15 days post inoculation. Disease was accessed 35 days following SCN inoculation. The interaction of *P. sojae* and SCN significantly affected soybean plant growth (Table 1). The *P. sojae* inoculated plants developed lesions on the roots and SCN inoculation did not show any visual symptoms in the soybean seedlings (Table 1, Table 2, Table 3, Table 4 and Table 5). In all the *P. sojae* treatments, the pathogen was isolated from the infected roots. However, *P. sojae* was not isolated from the soybean plants inoculated with SCN only and the non-inoculated checks.

#### 2.1.1. *P. sojae* Isolate PS-15-TF3 

A significant three-way cultivar × SCN × *P. sojae* interaction was observed to affect the stem length (χ2 = 151.7, df = 11, *p* < 0.001), root length (χ2 = 385.6, df = 11, *p* < 0.001), fresh plant weight (χ2 = 83.5, df = 11, *p* < 0.001) and fresh root weight (χ2 = 35.6, df = 11, *p* < 0.001) of the soybean plants (Table 1). In addition, there were significant two-way interactions observed between cultivar × SCN (*p* < 0.001), cultivar × *P. sojae* (*p* < 0.001) and *P. sojae* × SCN (*p* < 0.001) affecting stem length, root length, fresh plant weight and fresh root weight. While cultivar and *P. sojae* significantly affected all variables (*p* < 0.001), SCN significantly affected only root length (*p* = 0.01) and fresh plant weight (*p* = 0.02). 

#### 2.1.2. *P. sojae* Isolate PS-14-F14 

A significant three-way cultivar × SCN × *P. sojae* interaction was observed to affect all the measured growth parameters [stem length (χ2 = 116.4, df = 11, *p* < 0.001), root length (χ2 = 48.5, df = 11, *p* < 0.001), fresh plant weight (χ2 = 51.2, df = 11, *p* < 0.001)] except for fresh root weight (χ2 = 14.0, df = 11, *p* = 0.23) (Table 1). In addition, a significant two-way cultivar × SCN interaction (*p* < 0.001) affected stem length. A significant *P. sojae* × SCN interaction (*p* < 0.001) affected stem length, root length, and fresh plant weight. While *P. sojae* infection significantly affected only the stem length (*p* = 0.03) and SCN significantly affected stem length (*p* < 0.001), fresh plant weight (*p* < 0.001) and fresh root weight (*p* = 0.005), cultivar significantly affected all the variables except root length (*p* < 0.001).

### 2.2. Influence of P. sojae and SCN on Growth of Different Soybean Cultivars

#### 2.2.1. *P. sojae* Isolate PS-15-TF3

**Figure 1:**
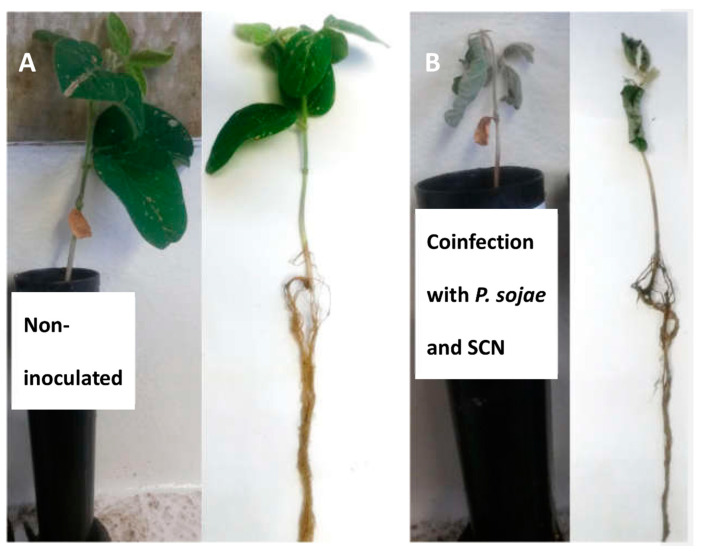
Phenotypic response of soybean plants (cv. Williams) at 35 days after inoculation following coinfection by *P. sojae* (PS-15-TF3) and SCN (**A**) non-inoculated (**B**) coinfection with *P. sojae* (Ps-15-TF3) and SCN.

Stem length of cv. Jack was significantly reduced in presence of SCN and *P. sojae* compared to non-inoculated control (8%; LSD = 11.4, *p* = 0.01). Likewise, stem length of the cv. Williams seedlings was reduced significantly when inoculated with *P. sojae* alone or in combination with SCN (14%; LSD = 12.5, *p* = 0.007). There was no stem reduction on the other two cultivars (cv. Surge and cv. William82) (Table 3 and Table 4). Significant root reduction was observed on cv. Surge (12%; LSD = 19.4, *P =* 0.00) and cv. Williams 82 (17%; LSD = 22.8, *p* = 0.04) and cv. Williams (19%; LSD = 28.9, *P =* 0.04) when infected by both the pathogens as compared to untreated controls (Table 3, Table 4 and Table 5). We observed significant fresh shoot reduction on cv. Surge (30%; LSD = 0.74, *P =* 0.005) and cv. Williams (60%; LSD = 0.56, *P =* 0.00) when soybean plants were inoculated with both SCN and *P. sojae* as compared to plants without SCN and *P. sojae* (Table 3 and Table 5; Figure 1). Similarly, significant drop of fresh root weight was observed on cv. Surge (42%; LSD = 0.46, *P =* 0.00) and cv. Williams (51%; LSD = 0.37, *P =* 0.00) in presence of SCN and *P. sojae* as compared to absence of both SCN and *P. sojae* (Table 3 and Table 5: Figure 1).

#### 2.2.2. *P. sojae* Isolate PS-14-F14

The reduction in stem length of cv. William 82 seedlings when inoculated with *P. sojae* alone or in combination with SCN (10%; LSD = 13.6, *p* = 0.04) approach significant in contrast with non-inoculated seedlings, but there was no reduction on other three cultivars. Presence of SCN and *P. sojae* greatly reduce the root growth on cv. Williams (18%; LSD = 28.9, *p* = 0.04) as opposed to absence of SCN and *P. sojae*. Moreover, Fresh shoot and root weight was significantly reduced on cv. Surge (32%; LSD = 0.74, *p* = 0.00; 40%; LSD = 0.46, *p* = 0.00) and cv. Williams (40%; LSD = 0.56, *p* = 0.00; 37%; LSD = 0.37, *p* = 0.00) when both pathogens inoculated as compared to untreated plants (Table 3, Table 4 and Table 5).

### 2.3. SCN Influence on P. sojae Pathotype

#### 2.3.1. *P. sojae* Isolate PS-15-TF3

A significant two-way cultivar × treatment (non-inoculated control, inoculated with SCN only, inoculated with PS-15- TF3 only, co-inoculation with PS-15-TF3 and SCN) interaction was observed to affect the lesion length caused by *P. sojae* (PS-15-TF3) on soybean (*p* < 0.001); therefore, lesion length data obtained for each cultivar was analyzed separately. The lesion size caused by PS-15-TF3 (complex pathotype) in presence of SCN was significantly higher (23%) on cv. Jack (LSD = 4.6, *p* < 0.001) compared to PS-15-TF3 alone. Similarly, due to coinfection longer lesion size was observed for cv. Surge, cv. William82 and cv. Williams, 15%, 10% and 8%, respectively, compared to PS-15-TF3 alone (Table 2, Table 3, Table 4 and Table 5).

#### 2.3.2. *P. sojae* Isolate PS-14-F14

A significant two-way cultivar × treatment (non-inoculated control, inoculated with SCN only, inoculated with PS-14-F14 only, co-inoculation with PS-14-F14 and SCN) interaction was observed to affect the lesion length caused by *P. sojae* on soybean (*p* < 0.001); therefore, lesion length data obtained for each cultivar was analyzed separately. Size of the lesion developed by PS-14-F14 (simple pathotype) in presence of SCN was not significantly higher in any of the cultivars used in this study indicating that presence of SCN did not influence soybean plant resistance against this *P. sojae* isolate (Table 2, Table 3, Table 4 and Table 5).

### 2.4. P. sojae Influence on SCN Development 

#### 2.4.1. *P. sojae* Isolate PS-15-TF3

A significant two-way cultivar × treatment interaction was observed to affect the SCN population on soybean plants (χ2 = 4.5, df = 1, *p* = 0.033); therefore, data obtained for SCN egg number was analyzed separately for each cultivar. Compared to SCN treatment only, the number of SCN eggs and juveniles was significantly reduced in the presence of PS-15-TF3 (complex pathotype) for all the four cultivars used in the study (Table 6). The highest 72% (LSD = 4423.3, *p* < 0.001) reduction was on cv. William82 followed by 50% (LSD = 1813.2, *p* < 0.001) on cv. Surge, 18% (LSD = 106.0, *p* = 0.025) on cv. Jack, respectively (Table 6). However, we did observe 16% SCN population reduction on cv. Williams (LSD = 446.7, *p* = 0.06) as compared to SCN treatment only but they did not differ statistically. 

#### 2.4.2. *P. sojae* Isolate PS-14-F14

A significant two-way cultivar × treatment (non-inoculated control, inoculated with SCN only, inoculated with PS-15-TF3 and SCN) interaction was observed to affect the SCN population on soybean seedlings (*p* < 0.001); therefore, data obtained for SCN egg numbers was analyzed separately for each cultivar. On cv. Surge and cv. William82, the number of SCN eggs and juveniles were significantly reduced by 69% (LSD = 1163.9, *p* < 0.001) and 47% (LSD = 4815.9, *p* < 0.001) in the presence of PS-14-F14 (simple pathotype) as compared to soybean plants inoculated with SCN only (Table 6). On the other two SCN resistant (cv. Jack) and SCN susceptible (cv. Willams) cultivars showed a non-significant reduction of SCN eggs and juveniles, 5% (LSD = 86.3, *p* = 0.37) and 8% (LSD = 493.0, *p* = 0.33), respectively, in presence of PS-14-F14 (simple pathotype) (Table 6).

## 3. Discussion

This study examined the interaction between two pathotypes of *P. sojae* and SCN on soybean in the greenhouse using the protocol established by Adenjii et.al. [18]. The *P. sojae* isolate PS-15-TF3 had a significant three-way cultivar × SCN × *P. sojae* interaction (*p* < 0.001) that was observed to affect the soybean plant health variables measured (stem length, root length, fresh plant weight and fresh root weight) in this study (Table 1). Additionally, there was a significant two-way *P. sojae* isolate PS-15-TF3 *×* SCN interaction (*p* < 0.001) observed affecting all the measured variables (Table 1). In contrast, *P. sojae* isolate PS-14-F14 had a significant three-way cultivar × SCN × *P. sojae* interaction (*p* < 0.001) that was observed to affect all the measured variables except fresh root weight of the soybean plants (Table 1). Similarly, there was a significant two-way *P. sojae* isolate PS-14-F14 × SCN interaction (*p* < 0.001) observed affecting all the measured variables except fresh root weight of the soybean plants. 

In our study, we observed noticeable effects on growth parameters when nematode and fungus were inoculated individually or in combination as compared to untreated control for both simple and complex pathotypes. For instance, on cv. Surge and cv. Williams root length and shoot and root weight declined significantly as compared to untreated control (Table 4 and Table 5). Adeniji et al. [18] reported similar observations that the shoot and root weight of three soybean cultivars (Carosoy, Dyer, and Harosoy-63) were lower when inoculated with *P. sojae* in combination with SCN compared to when inoculated with *P. sojae* alone, but the differences were not significant between these treatments. While comparing the effect of the two *P. sojae* pathotypes on the growth variables we observed that *P. sojae* isolate PS-14-F14 and its effect on growth variables was less as compared to that of isolate PS-15-TF3. For instance, coinfection caused by SCN and PS-15-TF3 (complex pathotype) reduce fresh shoot and root weight by 60 and 51% respectively, as compared to untreated control on cv William. Whereas coinfection of SCN and PS-14-F14 (simple pathotype) had fresh shoot and root weight reduction 40 and 37% respectively. Three of the cultivars used (cv. Jack, cv. Surge, and cv. William82) in our study had “isolate-specific resistance genes” only for *P. sojae* isolate PS-14-F14 indicating a reason for less effect on growth as relative to *P. sojae* inoculated resistance gene is still active. In the study by Mideros et al. [22], a significant isolate × host genotype interaction was observed for lesion length, infection frequency, and number of oospores, and it was speculated that the interaction was observed due to “isolate-specific resistance genes” since the two isolates they used varied in their virulence on the eight genotypes. 

While determining the effect of *P. sojae* pathotypes on soybean plant roots in the presence of SCN, we observed an increase in lesion size (by additive manner) in the range of approximately 8% to 23% due to PS-15-TF3 in the presence of SCN. Among the two *P. sojae* isolates, PS-15-TF3 (complex pathotype) was virulent on all 13 *Rps* differentials [1], and none of the four cultivars used in this study have resistance to this pathotype. Therefore, it might be speculated that lesion length caused by PS-15-TF3 increased in the presence of SCN because PS-15-TF3 was able to overcome the resistance in the three cultivars (Jack, Surge and Williams 82) despite the three cultivars having single *Rps* gene in their background. However, soybean cultivars when co-infected with PS-14-F14 and SCN, the lesion size remained unaffected indicating that the presence of SCN affected neither the *p*. *sojae* virulence nor alter the effectiveness of the *Rps* genes. Previous research on fungal-nematode interactions has shown that nematodes can wound plant roots and break-down resistance resulting in the plants being susceptible to fungal pathogens [15,19,23]. For example, greenhouse trials were conducted by Diaz [16] to determine whether SCN infestation enhances root rot caused by species of *Fusarium* on soybean using cultivars differing in genetic resistance to SCN. Two isolates from each of the eight *Fusarium* species used in the study were tested on root rot severity, the number of SCN females, and root morphological characteristics. Depending on the *Fusarium* isolates and species, enhanced root rot severity and root damage were observed when SCN was combined with the *Fusarium* isolates as compared to single pathogen treatment. Similar observations with regards to increased lesion length by *P. sojae* in the presence of SCN were made by Adeniji et al. [18] and Kaitany et al. [24] in the interaction study between the two pathogens. They hypothesized that SCN may be involved in modifying the physiology of soybean and thus increasing the susceptibility of the plants to infection by *P. sojae*. This is clearly in contrast with our study. The higher lesion length in the presence of SCN among the cultivars used in our study may be due to wounds and tissue damage by the nematodes [19] but the resistance of the *P. sojae* wasn’t necessarily compromised.

While studying the effect of *P. sojae* pathotypes on SCN, we observed that the nematode population was significantly reduced on three soybean cultivars (except cv. Williams) in the presence of the two *P. sojae* pathotypes (7% to 48%). In general, the ability of SCN to reproduce on soybean roots can be affected when the nematode cannot obtain nutrients at all from the host or cannot sustain feeding on the host because of the changes in the host’s defense mechanism [25]. In this study, a decrease in SCN population was observed on the soybean plants because the roots were infected and colonized by *P. sojae* and therefore, reduced uptake of nutrients and survival feeding cells for the nematode [18,19]. Moreover, *P. sojae* is known to produce toxic metabolites during the formation of sporangium that may affect the reproduction of SCN [26]. For example, in a study by Dong et al. [27], the expression of NLP protein (24-kDa protein that induces cell death and ethylene accumulation) in *P. sojae* was studied and it was shown that 20 of the NLP proteins were highly expressed during cyst germination and infection stages. Although the toxins produced by *P. sojae* were not explored in this study, it may be speculated that toxic metabolites produced by *P. sojae* may have affected the reproduction of SCN on soybean.

In summary, our study provides insight into the possible interaction between SCN and *P. sojae* on soybean under controlled conditions. Our results show that SCN and *P. sojae* do interact and thus compromise the overall growth variables of the soybean plants irrespective of the nature of virulence pathotypes. 

## 4. Materials and Methods

### 4.1. PhytoPhthora sojae Isolation, Identification and PathotyPe Characterization

For *P. sojae* inoculum, two isolates (designated as PS-15-TF3 and PS-14-F14) were recovered from soil samples collected from a commercial soybean field in Turner County (SD, USA) and in Bon Homme County (SD, USA), respectively [28]. 

To recover *P. sojae* isolates from the soil samples, a soil baiting method was used [29]. Styrofoam cups (473 mL, Draft Container Corporation, Mason, MI, USA) containing soil samples were flooded for 24 h using tap water, drained, and then air dried until the moisture content reached a matric potential of approximately −300 mb. The cups were placed in polyethylene bags and incubated at 22 °C for a total of 2 weeks. Following the incubation period, five seeds of the susceptible soybean cv. Williams (provided by Dr. Anne E. Dorrance, the Ohio State University, Columbus, OH, USA) were placed on top of the soil in the cups and covered with wet coarse vermiculite (Therm-O-Rock, New Eagle, PA, USA). Three days after planting of cv. Williams, the cups were flooded again for 24 h and then placed on greenhouse benches to drain the water. Ten days after planting, soybean seedlings were harvested; each seedling was rinsed under tap water, and then washed with antimicrobial soap (Equate, Bentonville, AR, USA) to remove soil off the plants [29]. After soil was removed, roots were kept under the running tap water for 30 min. Soybean roots were disinfested with 0.05% sodium hypochlorite for 30 s, washed in sterile distilled water and then air dried on a sterile paper towel. Small pieces of the symptomatic root (approximately 1 cm) were excised aseptically around the soil line and placed on the selective modified PBNIC medium (40 mL V8 juice (Campbell Soup Company, Camden, NJ, USA), 0.6 g CaCO_3_ (Sigma-Aldrich, St Louis, MO, USA), 0.2 g Bacto Yeast extract (Becton, Dickinson and Company, Erembodegem, Belgium), 1.0 g sucrose (Sigma-Aldrich), 20.0 g agar (Sigma-Aldrich) in 1000 mL distilled water) [30]. The PBNIC petri plates were incubated for 3 to 4 days at 22 ± 2 °C in dark. The plates were inverted to limit bacterial contamination. 

To purify *P. sojae* cultures, mycelial plugs were removed from the leading edges of colonies in the PBNIC plates and transferred to petri plates containing lima bean agar (100 mL lima bean broth and 20 g agar in 1000 mL distilled water: LBA). After 2 to 3 days of incubation at 22 °C and in dark, all the colonies were examined with a microscope (at 40× magnification) for characteristic appearance of mycelium and for oospore formation. The *P. sojae* isolates grew slowly on PBNIC agar media with dense white mycelium forming on the plates after 2 or 3 days. The mycelium appeared to be coenocytic, highly branched with curved tips on PBNIC media plates. The color of the hyphae was white and branched mostly at right angles [31]. After 3 days, mycelial plugs were removed from the leading edges of colonies and transferred to potato dextrose agar (PDA; Becton, Dickinson and Company, Franklin Lakes, NJ, USA) plates for the confirmation of *P. sojae*, since the pathogen does not grow on full strength PDA [32]. 

The identification of the two *P. sojae* isolates (PS-15-TF3 and PS-14-F14) was confirmed using the internal transcribed spacer (ITS) regions of ribosomal DNA [24]. DNA was extracted from the lyophilized mycelia of the two isolates grown in diluted V8 juice broth using the Wizard Genomic DNA Purification Kit (Promega Inc., Madison, WI, USA). The internal transcribed spacer (ITS) region of the DNA was amplified using ITS4 and ITS6 primers [33]. Reactions for the PCR amplifications were performed in a 20 μL mixture containing approximately 1–3 ng/μL of DNA, 400 nM of each the forward and reverse primers, 2 mM of each dNTPs, 5 units/μL of Taq DNA Polymerase (Qiagen, Valencia, CA, USA), and 10× Taq Buffer containing 15 mM MgCl_2_ (Qiagen). The PCR parameters included an initial denaturation at 94 °C for 3 min, followed by 35 cycles of denaturation at 94 °C for 1 min, annealing at 55 °C for 1 min, extension at 72 °C for 1 min, and a final extension at 72 °C for 10 min [33]. To confirm amplification, a 7 μL aliquot of both PCR products was run on an agarose gel (2%). The PCR products were sequenced by Functional Bioscience Inc. (Madison, WI, USA). Analysis of the edited ITS sequences of the two *P. sojae* isolates was performed using Basic Local Alignment Search Tool nucleotide (BLASTN) at GenBank nucleotide database (National Centre for Biotechnology Information, http://www.ncbi.nlm.nih.gov/, accessed on 20 July 2019). The two isolates were identified as *P. sojae* in the BLASTN searches based on lowest e-value (<10), highest score, and greatest similarity (>95%). Approximately 700 bp of the ITS region was amplified from the two *P. sojae* isolates and used to query the GenBank database. A BLASTN search matched the ITS sequence of the *P. sojae* isolates with the ITS sequence of *Phytophthora sojae* strain ATCC MYA-3899 (Accession # FJ746643) with identities = 837/838 (99%) and gaps = 0/838 (0%). The ITS sequences of the *P. sojae* isolates (PS-15-TF3 and PS-14-F14) generated in this study are deposited in the GenBank under accession numbers KX668417 and KX668418.

For the pathotype determination of the *P. sojae* isolates (PS-15-TF3 and PS-14-F14) the hypocotyl inoculation technique was adopted on a set of 13 soybean differentials [29] with each differential having one specific *Rps* gene. The 13 differentials used in this study were obtained from the USDA-ARS Soybean Germplasm Collection, Ohio State/OARDC and these included Harlon (*Rps*1a), Harosoy 13XX (*Rps*1b), Williams 79 (*Rps*1c), PI 103091 (*Rps* 1d),Williams 82 (*Rps*1k), L76-1988 (*Rps*2), L83-570 (*Rps*3a), PRX-146-36 (*Rps*3b), PRX-145-48 (*Rps*3c), L85-2352 (*Rps*4), L85-3059 (*Rps*5), Haro 62xx (*Rps*6), Harosoy (*Rps*7), PI 399073 (*Rps*8) [6]. The soybean cv. Williams was used a susceptible check. Fifteen seeds of 13 soybean differentials and cv. Williams were sown in each styrofoam cup (473 mL) and grown for 7 days at 25–28 °C under 16 h photoperiod with a light intensity of 1000 μEm^−2^s^−1^ in the greenhouse. During the 7 days, the plants were watered daily. To inoculate the differentials for pathotyping the two *P. sojae* isolates, a slurry was prepared from a 2-week-old culture of *P. sojae* grown on LBA. About 0.2 to 0.4 mL (approximately 200 to 400 cfu/mL) of the culture slurry was placed into the slit (1 cm) of the seedling’s hypocotyl region with the help of the syringe (10 mL). After inoculation, the plants were incubated in a dew chamber (95% humidity) for 24 h at a temperature range of 20 to 22 °C in the dark. After 24 h of incubation, the soybean plants were placed in a greenhouse at temperatures ranging from 22 to 28 °C under natural light. Five to seven days after inoculation, the incidence of *Phytophthora* root rot was evaluated [29]. The differential was considered susceptible when at least 7 of the 10 seedlings developed an expanding necrotic brown lesion. A differential was considered resistant if 70% or more of the plant inoculated with *P. sojae* survived [29]. Based on the reaction of *P. sojae* isolates on the soybean differential, the Octal Code was determined with HaGiS spread sheet as described by Herrmann et al. [9]. The *P. sojae* isolate PS-15-TF3 caused susceptible reaction to all the 13 (complex) soybean differentials (*Rps*1a, *Rps*1b, *Rps*1c, *Rps*1d, *Rps*1k, *Rps*2, *Rps*3a, *Rps*3b, *Rps*3c, *Rps*4, *Rps*5, *Rps*6 and *Rps*7) and is represented by virulence formula 77771. The *P. sojae* isolate PS-14-F14 showed susceptible reaction to only one (simple) soybean differential (*Rps*7) and is represented by virulence formula 00001 (formally Race 1).

### 4.2. SCN Extraction and Inoculum 

For SCN inoculum, eggs, and juveniles of *H. glycines* were recovered from a soil sample collected from Clay County (SD, USA) and the population was determined to be HG type 0 in a study conducted by Acharya et al. [34]. In this study, *H. glycines* HG type 0 was used because it was identified as the most common HG type on soybean in South Dakota by Acharya et al. [34].

To increase SCN population for the interaction study, population of *H. glycines* were increased on the SCN susceptible cv. Williams 82 in a water bath set at a temperature of 26 ± 2 °C in the greenhouse (air temperature of 25 ± 2 °C in the greenhouse, with natural light supplements with a photoperiod of 16 h of artificial light for 35 days). The cysts of HG type 0 were collected in a 50 mL beaker using the method described by Faghihi et al. [35]. Cysts were crushed and SCN eggs were released from cysts with a stopper –bit assembly [36]. The nematode inoculum was prepared in a deionized water suspension by counting (average of three counts) 2000 SCN eggs and juveniles (per ml) using a nematode counting slide under a dissecting microscope at 40× magnification (Nikon SMZ745T, Nikon Instruments, ON, Canada). 

### 4.3. Interaction between P. sojae and SCN

For the interaction study between *P. sojae* and SCN, the experiment was set up in a completely randomized design in a factorial arrangement for the two *P. sojae* isolates, PS-15-TF3 and PS-14-F14, in the greenhouse. Four soybean cultivars (Jack, Surge, Williams 82, and Williams) were used for the study which differed in their resistance to SCN and *P. sojae* [Jack is resistant to SCN and has *Rps*2 gene conferring tolerance to *P. sojae* [37]; Surge has *Rps*1 gene conferring resistance to *P. sojae* and susceptible to SCN [38]; Williams 82 is SCN susceptible [39] and has *Rps*1k gene conferring resistance to *P. sojae* [6]; Williams is susceptible to SCN [40] and susceptible to *P. sojae* [6]. For each *P. sojae* isolate, there were 4 treatments (SCN only, *P. sojae* only, concomitant inoculation of SCN and *P. sojae* and non-inoculated control) and 5 replicates per treatment on all 4 soybean cultivars. Each plant in a cone-tainer was regarded as a replication. The experiment was performed twice. 

Before planting in 164 mL cone-tainers (Stuewe and Sons Inc., Tangent, OR, USA), the seeds of the 4 soybean cultivars were pre-germinated in petridishes for 3 days. For each cultivar, a total of 30 cone-tainers were filled with 80 g of steam-pasteurized sand: soil (silty clay loam; [41] (2 parts of sand: 1 parts of soil) mixture. Two agar plugs (5 mm diameter) from 10-day old LBA cultures of *P. sojae* were placed on either side of the pre-germinated soybean seeds at 10 mm [18]. The *P. sojae* inoculum was covered with 20 g of the steam pasteurized sand: soil (silty clay loam; [41] mixture (2:1). After inoculating the soybean plants with either of the *P. sojae* isolates, the plants were transferred into a misting chamber for 48 h before SCN inoculation. After 48 h, a 25 mm deep hole was carefully made close to the soybean seedlings in each of the cone-tainers needing SCN treatment using a glass rod (0.5 mm diameter) and 1 mL of the SCN suspension (containing 2000 eggs and juveniles) were added to the holes [17]. The cone-tainers were placed in buckets filled with sand and maintained in a water bath at 26 ± 2 °C in the greenhouse, with natural light supplements with a photoperiod of 16 h of artificial light for 35 days. The relative humidity in the greenhouse was maintained at 95% and air temperature was set at 22 to 25 °C. The containers were watered every other day with 25 mL of tap water. To ensure of getting all the soybean roots (roots might have grown out of the bottom of the containers) the bottom of the containers were raped with 127 mm × 127 mm of square pieces of 1.5-Ounce Weed-Barrier Fabric (DeWitt, Sikeston, MO, USA) with the help of rubber bands.

At 35 days after SCN inoculation, data were collected on stem length, root length, fresh plant weight, fresh root weight, lesion length produced by *P. sojae* on soybean roots, number of SCN eggs and juveniles per plant for each treatment. The lesion length caused by *P. sojae* was measured from the site of root initiation to the end of the main soybean roots where the lesion would have extended on each soybean seedling (modified from Mideros et al. [22]. For the extraction of SCN cysts from the roots of the seedlings the cone-tainers were taken out of the bucket and uprooted gently after soaking with water for 15 min. The SCN females were dislodged from the soybean roots by spraying with a strong stream of water. The females were collected 40 in a 250-µm-pore sieve nested under a 710-µm-pore sieve. Cysts were crushed, SCN eggs were released from cysts [36] and finally eggs, and juveniles were counted using a nematode counting slide under a dissecting microscope at 40× magnification (Nikon SMZ745T, Nikon Instruments, ON, Canada). 

To confirm pathogenicity of *P. sojae*, infected roots of random soybean plants representing *P. sojae* treatments (*P. sojae* only and concomitant inoculation of SCN and *P. sojae*) were sectioned longitudinally (approximately 1 cm length), surface-sterilized and placed on LBA. Plates were incubated at 22 °C for 2 to 3 days in the dark and cultures were scored for presence or absence of *P. sojae* based on morphology [31]. 

### 4.4. Data Analysis

To determine whether the interaction between SCN and *P. sojae* can affect soybean growth, the relationship between soybean cultivars, *P. sojae* and SCN infestation was analyzed using the linear mixed effects models in R (v2.11.1; R core team 2012; https://www.rstudio.com/, accessed on 9 January 2019) using the *lme4* [42] package. For the model, the experimental factors “cultivar (Jack, Surge, Williams 82, and Williams)”, “*P. sojae* infestation (infested soybean roots or not)” and “SCN infestation (infested soybean roots or not)” were entered as fixed effects. “experimental repeat” and “replication” were included as random effects into the model. Stem length, root length, fresh plant weight and fresh root weight were considered as dependable variables. The *p*-values associated with the growth variables (stem length, root length, fresh root weight, fresh plant weight) was determined using the likelihood ratio test [in the *lme4* package [42] in which a “full” model containing fixed effects and random effects was compared against a “reduced” model with only random effects. For the likelihood ratio test, the fixed effects were considered significant if the difference between the likelihood of the full model and reduced model was significant at *p* ≤ 0.05. 

To determine the influence of *P. sojae* and SCN on soybean growth, the effect of *P. sojae* on SCN or the effect of SCN on *P. sojae*, the treatment effect was analyzed using the linear mixed effects models in R using the *lme4* [42] package. For the model, the experimental factors “cultivar” and “treatment (non-inoculated control, *P. sojae* only or SCN only or concomitant inoculation of the two pathogens)” were entered as fixed effects. As random effects, “experimental repeat” and “replication” were included into the model. Growth parameters (stem length, root length, fresh plant weight and fresh root weight), lesion length and SCN eggs and juvenile counts were considered as dependable variable for the analysis. In addition, growth parameters (stem length, root length, fresh plant weight and fresh root weight), the lesion length caused by *P. sojae* pathotypes and SCN eggs and juvenile counts were subjected to analysis of variance (ANOVA) for a completely randomized design in R and treatment means were separated using Fisher’s LSD test (*p* ≤ 0.05) in the Agricolae package version 1.3-5 [28]. 

For all analyses, the ANOVA assumptions of normality and homogeneity of variances were checked and satisfied before combining the results of the two experimental repeats. 

## 5. Conclusions

In general, the interaction between multiple pests on soybean can lead to higher yield losses under field conditions. For example, field studies were conducted by Diaz [11] on the interaction between SCN and *Fusarium* root rot species affecting root rot severity and enhanced yield losses was observed to be higher in the combined presence of SCN and *Fusarium*. For this study, we did not test the effect of interaction between *P. sojae* and SCN on soybean under field conditions. However, it is possible that yield and other agronomic factors can be compromised because of the interaction between the two pathogens. Currently, *P. sojae* and SCN are managed using integrated disease management approaches such as selecting soybean varieties with tolerance to *P. sojae* and resistance to SCN, seed treatments and crop rotation. Based on our results, infection of soybean plants by *P. sojae* may be exacerbated by SCN depending on the nature of pathotypes that exist in the farmers’ fields. We, therefore, recommend soybean farmers to use cultivars with durable resistance to SCN and *P. sojae* to manage the disease complex and reduce yield loss in their fields.

## Figures and Tables

**Table 1:** *p* values for main and interaction effect of cultivars (Jack, Surge, William 82, and Williams) and pathogen treatments [SCN, *P. sojae* isolates (PS-15-TF3, PS-14-F14) or concomitant inoculations of the two pathogens] on soybean growth.

*P. sojae* Isolates	Effects ^a,b,c,d^							
	Variables	Cultivar	SCN	*P. sojae*	Cultivar × SCN	Cultivar × *P. sojae*	SCNx *P. sojae*	Cultivar × SCN × *P. sojae*
**PS-15-TF3**	Stem length	<0.001	ns	0.008	0.02	ns	<0.001	<0.001
	Root length	<0.001	0.01	<0.001	<0.001	ns	<0.001	<0.001
	Fresh plant weight	<0.001	0.02	0.001	0.003	0.03	<0.001	<0.001
	Fresh root weight	<0.001	ns	0.002	0.014	ns	<0.001	<0.001
**PS-14-F14**	Stem length	<0.001	0.03	0.004	0.025	ns	<0.001	<0.001
	Root length	<0.001	ns	ns	ns	ns	<0.001	<0.001
	Fresh plant weight	<0.001	<0.001	ns	ns	ns	<0.001	<0.001
	Fresh root weight	ns	0.005	ns	ns	ns	ns	ns

^a^*p*-values associated with growth variables (stem length, root length, fresh root weight, and fresh plant weight) was determined using the likelihood ratio test [in the *lme4* [5] package] in which a “full” model containing fixed effects was compared against a “reduced” model without the fixed effects. For the likelihood ratio test, the fixed effect was considered significant if the difference between the likelihood of the full and reduced models was significant at *p* ≤ 0.05. ^b^ Analysis of variance was conducted by combining the data of two experimental repeats after testing for homogeneity of variance at *p* ≤ 0.05 in R. ^c^ ns = not significant at *p* ≥ 0.05. ^d^ Abbreviation: SCN = Soybean Cyst Nematode.

**Table 2:** Shoot, root length and weight, lesion length measurements observed on the soybean cv. Jack inoculated with SCN or concomitant inoculations of SCN with either of the *P. sojae* isolates (PS-15-TF3 and PS-14-F14).

*P. sojae* Isolates	Treatments ^a,b,c^	Stem Length (mm) ^a^	Root Length (mm) ^a^	Fresh Plant Weight (g) ^a^	Fresh Root Weight (g) ^a^	Lesion Length (mm) ^d^
	Non treated control	177.5 a	211.0 a	3.3 b,c	1.8 a,b	N/A
	SCN	164.3 b,c	208.7 a	3.2 b,c	1.7 a,b	N/A
PS-15-TF3	PS-15-TF3	173.7 a,b	214.3 a	4.1 a	1.9 a	60.6 b
	PS-15-TF3 + SCN	162.5 b,c	205.0 a	3.2 b,c	1.5 b	78.7 a
PS-14-F14	PS-14-F14	165.6 b,c	217.5 a	3.8 a,b	1.7 a,b	36.9 c
	PS-14-F14 + SCN	158.7 c	213.1 a	3.3 b,c	1.6 a,b	43.1 c

^a^ Analysis of variance was conducted by combining the data of two experimental repeats after testing for homogeneity of variance at *p* ≤ 0.05 in R. Values are the means of two experiments with a total of ten replications each. Values within a column followed by the same letter are not significantly different according to Fisher’s least significant difference (*p* ≤ 0.05). ^b^ Treatments involving PS-15-TF3 include those treatments that were non inoculated control (five replications), inoculated with only PS-15-TF3 (five replications), SCN inoculation only (five replication) and concomitant inoculation with SCN (five replications). ^c^ Treatments involving PS-14-F14 include those treatments that were non inoculated control (five replications), inoculated with only PS-14-F14 (five replications), SCN inoculation only (five replication) and concomitant inoculation with SCN (five replications). ^d^ Lesion length caused by *P. sojae* was measured from the site of seed attachment to the end of the soybean roots where the lesion would have extended on each soybean seedling (Modified from Mideros [22]). On the SCN control, no lesion was observed on the soybean roots and no pathogen was recovered.

**Table 3:** Shoot, root length and weight, lesion length measurements observed on the soybean cv. Surge inoculated with SCN or concomitant inoculations of SCN with either of the *P. sojae* isolates (PS-15-TF3 and PS-14-F14).

*P. sojae* Isolates	Treatments ^a,b,c^	Stem Length (mm) ^a^	Root Length (mm) ^a^	Fresh Plant Weight (g) ^a^	Fresh Root Weight (g) ^a^	Lesion Length (mm) ^d^
	Non treated control	182.5 a	230.6 a	4.1 a	2.6 a	N/A
	SCN	181.2 a	225.0 b	3.4 a,b	1.7 b	N/A
PS-15-TF3	PS-15-TF3	180.0 a	226.7 b	3.2 b	1.7 b	35.0 b
	PS-15-TF3 + SCN	173.1 a	201.8 b	2.9 b	1.5 b	41.2 a
PS-14-F14	PS-14-F14	175.6 a	220.0 b	2.8 b	1.8 b	37.5 b
	PS-14-F14 + SCN	171.8 a	212.5 b	2.8 b	1.6 b	38.1 b

^a^ Analysis of variance was conducted by combining the data of two experimental repeats after testing for homogeneity of variance at *p* ≤ 0.05 in R. Values are the means of two experiments with a total of ten replications each. Values within a column followed by the same letter are not significantly different according to Fisher’s least significant difference (*p* ≤ 0.05). ^b^ Treatments involving PS-15-TF3 include those treatments that were non inoculated control (five replications), inoculated with only PS-15-TF3 (five replications), SCN inoculation only (five replication) and concomitant inoculation with SCN (five replications).^c^ Treatments involving PS-14-F14 include those treatments that were non inoculated control (five replications), inoculated with only PS-14-F14 (five replications), SCN inoculation only (five replication) and concomitant inoculation with SCN (five replications). ^d^ Lesion length caused by *P. sojae* was measured from the site of seed attachment to the end of the soybean roots where the lesion would have extended on each soybean seedling (Modified from Mideros et al. [22]). On the SCN and non-inoculated control, no lesion was observed on the soybean roots and no pathogen was recovered.

**Table 4:** Shoot, root length and weight, lesion length measurements observed on the soybean cv. William 82 inoculated with SCN or concomitant inoculations of SCN with either of the *P. sojae* isolates (PS-15-TF3 and PS-14-F14).

*P. sojae* Isolates	Treatments ^a,b,c^	Stem Length (mm) ^a^	Root Length (mm) ^a^	Fresh Plant Weight (g) ^a^	Fresh Root Weight (g) ^a^	Lesion Length (mm) ^d^
	Non treated control	176.8 a,b	221.2 a	3.0 a,b	1.7 a	N/A
	SCN	185.6 a	209.2 a,b	3.3 a	1.9 a	N/A
PS-15-TF3	PS-15-TF3	178.9 a,b	199.2 b,c	2.6 b	1.7 a	63.1 b
	PS-15-TF3 + SCN	177.5 a,b	184.3 c	2.6 b	1.7 a	70.0 a
PS-14-F14	PS-14-F14	180.0 a	208.7 a,b	3.5 a	1.9 a	60.6 b
	PS-14-F14 + SCN	166.2 a,b	198.7 a,b,c	3.3 a	1.7 a	64.1 b

^a^ Analysis of variance was conducted by combining the data of two experimental repeats after testing for homogeneity of variance at *p* ≤ 0.05 in R. Values are the means of two experiments with a total of ten replications each. Values within a column followed by the same letter are not significantly different according to Fisher’s least significant difference (*p* ≤ 0.05). ^b^ Treatments involving PS-15-TF3 include those treatments that were non inoculated control (five replications), inoculated with only PS-15-TF3 (five replications), SCN inoculation only (five replication) and concomitant inoculation with SCN (five replications). ^c^ Treatments involving PS-14-F14 include those treatments that were non inoculated control (five replications), inoculated with only PS-14-F14 (five replications), SCN inoculation only (five replication) and concomitant inoculation with SCN (five replications). ^d^ Lesion length caused by *P. sojae* was measured from the site of seed attachment to the end of the soybean roots where the lesion would have extended on each soybean seedling (Modified from Mideros et al. [22]). On the SCN and non-inoculated control, no lesion was observed on the soybean roots and no pathogen was recovered.

**Table 5:** Shoot, root length and weight, lesion length measurements observed on the soybean cv. Williams inoculated with SCN or concomitant inoculations of SCN with either of the *P. sojae* isolates (PS-15-TF3 and PS-14-F14).

*P. sojae* Isolates	Treatments ^a,b,c^	Stem Length (mm) ^a^	Root Length (mm) ^a^	Fresh Plant Weight (g) ^a^	Fresh Root Weight (g) ^a^	Lesion Length (mm) ^d^
	Non treated control	130.0 a	224.2 a	3.6 a	2.6 a	N/A
	SCN	130.0 a	195.0 b	2.3 b	1.7 b	N/A
PS-15-TF3	PS-15-TF3	115.0 b,c	191.2 b	1.7 c,d	1.3 c,d	75.6 b
	PS-15-TF3 + SCN	111.2 c	181.2 b	1.6 d	1.2 d	81.9 a
PS-14-F14	PS-14-F14	130.0 a	187.5 b	2.5 b	1.8 b	36.6 c
	PS-14-F14 + SCN	125.0 a,b	183.7 b	2.2 b,c	1.6 b,c	40.6 c

^a^ Analysis of variance was conducted by combining the data of two experimental repeats after testing for homogeneity of variance at *p* ≤ 0.05 in R. Values are the means of two experiments with a total of ten replications each. Values within a column followed by the same letter are not significantly different according to Fisher’s least significant difference (*p* ≤ 0.05). ^b^ Treatments involving PS-15-TF3 include those treatments that were non inoculated control (five replications), inoculated with only PS-15-TF3 (five replications), SCN inoculation only (five replication) and concomitant inoculation with SCN (five replications). ^c^ Treatments involving PS-14-F14 include those treatments that were non inoculated control (five replications), inoculated with only PS-14-F14 (five replications), SCN inoculation only (five replication) and concomitant inoculation with SCN (five replications). ^d^ Lesion length caused by *P. sojae* was measured from the site of seed attachment to the end of the soybean roots where the lesion would have extended on each soybean seedling (Modified from Mideros et al. [22]). On the SCN and non-inoculated control, no lesion was observed on the soybean roots and no pathogen was recovered.

**Table 6:** Mean number of SCN eggs (per gm of soybean root weight) on each of the four soybean cultivars from treatments.

Cultivar	*P. sojae* Isolates	Treatments ^a,b^	SCN (Per gm of Soybean Root Weight) ^c,d^
Jack	Non-inoculated control	N/A	
	PS-15-TF3	SCN	806.1 a
		PS-15-TF3 + SCN	682.7 b
	PS-14-F14	SCN	806.1 a
		PS-14-F14 + SCN	768.9 a
Surge	Non-inoculated control	N/A	
	PS-15-TF3	SCN	19680.2 a
		PS-15-TF3 + SCN	13116.2 b
	PS-14-F14	SCN	19680.2 a
		PS-14-F14 + SCN	11577.4 b
Williams 82	Non-inoculated control	N/A	
	PS-15-TF3	SCN	30811.5 a
		PS-15-TF3 + SCN	16044.8 b
	PS-14-F14	SCN	30811.5 a
		PS-14-F14 + SCN	17730.2 b
Williams	Non-inoculated control	N/A	
	PS-15-TF3	SCN	3081 a
		PS-15-TF3 + SCN	2660 a
	PS-14-F14	SCN	3081 a
		PS-14-F14 + SCN	2853 a

^a^ Treatments involving PS-15-TF3 include all those that were inoculated with SCN (five replications) and concomitant inoculation with SCN (five replications). The LSD analyses was performed by cultivar for treatments involving PS-15-TF3. ^b^ Treatments involving PS-14-F14 include all those that were non inoculated control (five replications), inoculated with SCN (five replications) and concomitant inoculation with SCN (five replications). The LSD analyses was performed by cultivar for treatments involving PS-15-TF3. ^c^ Analysis of variance was conducted by combining the data of two experimental repeats after testing for homogeneity of variance at *p* ≤ 0.05 in R. Values are the means of two experiments with a total of ten replications each. Values within a column followed by the same letter are not significantly different according to Fisher’s least significant difference (*p* ≤ 0.05). ^d^ SCN eggs and juveniles were counted using a nematode counting slide under a dissecting microscope at 40× magnification (Nikon SMZ745T, Nikon Instruments, ON, Canada). On the *P. sojae* control, no SCN eggs was observed under the microscope.

## Data Availability

Not applicable.
